# Young Women and Myocardial Infarction: Unveiling Clinical Patterns and Prognostic Outcomes

**DOI:** 10.7759/cureus.71865

**Published:** 2024-10-19

**Authors:** Fares Azaiez, Fekher Jaoued, Rami Tlili, Rim Ben Romdhane, Lagha Elyes, Meriem Drissa, Youssef Ben Ameur

**Affiliations:** 1 Cardiology Department, Mongi Slim Hospital, Tunis, TUN

**Keywords:** myocardial infarction, prognosis, revascularization, women, young

## Abstract

Background

Myocardial infarction (MI) remains a critical emergency with an increasing incidence among young women exposed to various risk factors. Despite extensive data on MI, there is limited information on premature coronary artery disease in women under the age of 50 years.

This study describes the clinical, paraclinical, and angiographic characteristics of MI in young women compared to older women and determines the prognosis.

Methods

This is a single-center retrospective study including women hospitalized between July 2019 and December 2021 in the cardiology department for the evaluation of MI. The population was divided into two groups based on age: women under 50 years, classified as young women, and those aged 50 years and above. A comparison was made between these two groups.

Results

A total of 197 women were included in our study. Forty-four women under 50 and 153 over 50 years were included. The mean age of young women was 44 ± 6 years. The main cardiovascular (CV) risk factors in young women were smoking (46%), hypertension (53%), diabetes (44%), and family history of coronary artery disease (21%). Autoimmune disease was present in 11%. Of the young women, 34% were admitted for ST-elevation myocardial infarction (STEMI), with 47% consulting late (>12 hours). The majority (91%) presented with typical chest pain. Monovessel disease was observed in 57% of young women. The left anterior descending artery was the most affected at 55%. Atherosclerosis was the most noted etiology (66%), followed by spontaneous coronary artery dissection (SCAD) (16%).

The comparative study showed that young women had fewer overall CV risk factors but a higher prevalence of smoking, familial history of coronary artery disease, and autoimmune disease. Young women presented more frequently with non-ST elevation myocardial infarction (NSTEMI). Monovessel disease was more common, and they required less myocardial revascularization by percutaneous intervention. Young women presented more with SCAD and less with atherosclerotic MI.

The in-hospital follow-up showed that young women experienced fewer major cardiac and cerebrovascular events (MACCE) compared to older women, with no in-hospital deaths recorded among young women. Long-term follow-up revealed a lower incidence of MACCE among young women (11% vs. 32.7% in older women) and a similar low mortality rate. Survival analysis showed that young women had a longer event-free survival time for MACCE (91.3 months) compared to older women (65.5 months).

Conclusions

The incidence of MI in young women is increasing. Smoking and hypertension are major risk factors. Hospital complications are rare, and prognosis is generally good, with low mortality rates.

## Introduction

As a result of lifestyle, eating habits, and physical inactivity, the incidence of cardiovascular disease in the young population, especially young women, is currently increasing [1].

Myocardial infarction (MI) in older women appears to share the same risk factors and pathophysiology as in men [2]. However, coronary disease in young women differs from that in older women in etiologies and prognosis as many cases may be of non-atherosclerotic origin [3]. There is currently a lack of studies to draw definitive conclusions for young women.

The aim of this study was to identify the clinical and angiographic particularities as well as the prognosis of MI in young women compared to older women.

## Materials and methods

Study population

We conducted a retrospective, analytical, descriptive, and monocentric study, including all women hospitalized for MI over a period of 30 months from July 1, 2019, to December 31, 2021. Eligible women presented evidence of type I MI following the Fourth Universal Definition of Myocardial Infarction by the European Society of Cardiology (ESC) [4].

The exclusion criteria for the study included women who were unable to provide informed consent for coronary angiography and/or angioplasty. Additionally, women who had not undergone coronary angiography were also excluded from participation in the study.

Data collection and variables

All women were examined on admission by trained medical staff. Data were collected from patient files. Information about age and cardiovascular risk factors (CVRF) (hypertension, diabetes mellitus, smoking, dyslipidemia, family history of cardiovascular disease, and obesity (BMI ≥ 30 kg/m²)) was collected. Medical history data included prior stroke, chronic kidney disease (glomerular filtration rate (GFR) ≤ 60 ml/min/1.73 m²), chronic lung disease, psychiatric disorders, and history of autoimmune disease. Gynecological data of the patients were also collected, including menopausal status, age of menopause if postmenopausal, and data on replacement therapy (oral contraception or hormone replacement therapy). Clinical characteristics of the MI at presentation were also obtained.

All patients had a complete clinical examination and biological assessment, including ultra-sensitive troponin, hemoglobin (anemia for a value less than 12 g/dl), platelets, leukocytes, markers of inflammation (C-reactive protein (CRP)), and renal function (creatinine), and a set of invasive and non-invasive complementary examinations, including electrocardiogram, echocardiography, cardiac MRI, if needed, and coronary angiography. The angiographic parameters collected were the presence of a significant lesion (any obstruction of at least 50% of the diameter of the main coronary trunks), the type of coronary obstruction, the site of the obstruction lesion, and the coronary status. In the case of spontaneous coronary artery dissection (SCAD), the site and type of SCAD were classified following the angiogram classification by Saw [5].

For the in-hospital evolution, all complications that occurred during hospitalization were recorded. Patients were systematically reviewed at the outpatient clinic one month after discharge and subsequently at variable intervals depending on their clinical situation. We specified for each patient their evolutionary progress and the occurrence of major cardiac and cerebrovascular events (MACCE). Our primary endpoint was the occurrence of all-cause mortality during the follow-up period.

Statistical analysis

The population was divided into two groups: women aged below 50 years and women over 50 years. We opted for this age limit because it has been used in several studies [6,7], thus allowing analytical study and decreasing the possibility of any impact related to menopausal hormonal imbalances, which might affect MI incidence in this population. This would facilitate the comparison of our results with those found in the literature. Univariate analysis was performed to evaluate the differences between the two age groups across various clinical variables and outcomes.

The data were analyzed using SPSS version 26 software (IBM Corp., Armonk, NY). Qualitative variables were summarized by absolute frequencies and percentages, while quantitative variables were summarized by means and standard deviations for normally distributed values, and by medians and interquartile ranges when the distribution was not normal. Comparisons between categorical variables were made using the chi-square (χ²) test, and differences between continuous variables were assessed using the t-test. For all statistical tests, the significance level was set at 0.05. Survival data without cardiovascular events were analyzed by establishing survival curves using the Kaplan-Meier method.

## Results

A total of 197 patients were evaluated. We divided the women hospitalized for MI into two groups: 44 young women between 18 and 50 years old, and 153 older women over 50 years old.

Sociodemographic data and cardiovascular risk factors

The mean age of the young women group was 44 years old ± 6 years. At least one CVRF was found in 91% of the patients in this group. Only four women (9%) did not present any CVRF. We noticed that there were significantly more smokers among young women compared to older women (46% vs. 19.6). Family history and the presence of an autoimmune disease were significantly associated with the occurrence of MI in young women. Concerning the gynecological data, only two young women (4.5%) and one older woman (0.7%) were on oral contraceptive pills. Baseline characteristics of the study groups are presented in Table 1.

**Table 1:** Comparison of cardiovascular risk factors, comorbidities, and clinical presentations. CAD: coronary artery disease; MI: myocardial infarction; NSTEMI: non-ST elevation myocardial infarction; STEMI: ST-elevation myocardial infarction.

	Group ≤ 50 years old (N = 44)	Group > 50 years old (N = 153)	p-value
Age (years)	44 ± 6	66.9 ± 8	<0.001
Cardiovascular risk factors			
Hypertension	23 (53%)	110 (71.9%)	0.014
Diabetes	19 (44%)	102 (67%)	0.005
Dyslipidemia	10 (23%)	43 (28.1%)	0.478
Smoking	20 (46%)	30 (19.6%)	0.001
Obesity	11 (25%)	30 (19.6%)	0.437
Family history of CAD	9 (21%)	7 (4.6%)	0.001
Comorbidities			
Ischemic stroke	1 (2%)	14 (9.2%)	0.198
Peripheral artery disease	1 (2%)	1 (0.7%)	0.345
Autoimmune disease	5 (11%)	0 (0%)	<0.0001
Chronic lung disease	4 (9%)	5 (5.3%)	0.345
Chronic kidney disease	1 (2%)	6 (4%)	0.603
Psychiatric disorder	1 (2%)	4 (2.6%)	0.899
Gynecological data			
Menopausal	12 (27%)	149 (97.4%)	<0.001
Age of menopause (years old)	46 ± 3	48.9 ± 2.9	0.089
Early menopause	6 (14%)	11 (7.2%)	0.150
Clinical presentations			
Chest pain	43 (98%)	144 (94.1%)	0.463
Typical chest pain	40 (91%)	128 (83.7%)	0.62
Dyspnea	1 (2%)	23 (15%)	0.001
Vomiting	2 (5%)	4 (2.6%)	0.617
Palpitations	2 (5%)	3 (2%)	0.311
Syncope	2 (5%)	6 (4%)	0.567
Type of MI			
STEMI	15 (34%)	54 (35.3%)	0.022
STEMI <12 h	8 (18%)	33 (21.6%)	0.169
STEMI >12 h	7 (16%)	21 (13.7%)	0.169
STEMI >6 h	14 (32%)	46 (30.1%)	0.672
NSTEMI	29 (66%)	99 (64.7%)	0.022
Very high risk	22 (50%)	71 (46.4%)	0.177
High risk	7 (16%)	28 (18.3%)	0.134
Physical examination findings			
Left heart failure	3 (5%)	36 (23.5%)	0.009
Right heart failure	0 (0%)	1 (0.7%)	0.304
Global heart failure	1 (2%)	11 (7.2%)	0.306
Cardiogenic shock	0 (0%)	9 (5.9%)	0.256

The diagnosis, clinical presentations, and clinical examination data

The majority of young women (29, 66%) were admitted for ST-elevation myocardial infarction (STEMI) while 15 (34%) were admitted for non-ST elevation myocardial infarction (NSTEMI). Interestingly, the proportion of young women admitted for STEMI was lower than that of older women (34% vs. 35.3%, p = 0.022). Additionally, the majority of young women (93%) presented late, more than six hours after the onset of symptoms (93% vs. 85.18%, p = 0.672). The majority of young women were in a good overall state upon admission, which is reflected by the lower incidence of left heart failure compared to the older group.

ECG data analysis and transthoracic echocardiography results

All young women showed sinus rhythm upon admission, while 4.6% of older women presented with atrial fibrillation at admission. Interestingly, a significant preponderance of normal ECG was noted among young women (11% vs. 3.3%). Notably, an anterior MI was significantly less prevalent among young women compared to their older counterparts, with no significant disparities observed in other MI localizations (Table 2).

**Table 2:** Comparison of electrocardiogram and transthoracic echography findings. ECG: electrocardiogram; LVEDD: left ventricular end-diastolic diameter; LVEF: left ventricular ejection fraction; LVESD: left ventricular end-systolic diameter; MI: myocardial infarction; RV: right ventricle; TAPSE: tricuspid annular plane systolic excursion.

	Group ≤ 50 years old (N = 44)	Group > 50 years old (N = 153)	p-value
Rhythm			
Sinus rhythm	44 (100%)	146 (95.4%)	0.352
Atrial fibrillation	0 (0%)	7 (4.6%)	0.352
Normal ECG	5 (11%)	5 (3.3%)	0.005
MI localization			
Anterior wall	8 (18%)	27 (17.6%)	0.009
Inferior wall	6 (15%)	20 (13%)	0.120
Other	1 (2%)	18 (11.7%)	0.407
Left ventricle dimension			
LVEDD (mm)	46 ± 4	47.5 ± 6.6	0.275
LVESD (mm)	28 ± 4	28.4 ± 2.9	0.089
LVEF (%)	53 ± 10	51.1 ± 12.5	0.342
Reduced LVEF	8 (18%)	38 (24.8%)	0.922
Midrange LVEF	11 (25%)	23 (15%)	0.174
Preserved LVEF	25 (57%)	90 (58.8%)	0.109
RV parameters			
S’ (cm/s)	11 ± 1	12.1 ± 1.8	0.404
TAPSE (mm)	20 ± 2	21.3 ± 2.9	0.419
RV dysfunction	6 (14%)	11 (3.9%)	0.230

No differences were observed between the two groups in terms of LVEF. Furthermore, a tendency toward right ventricular dysfunction was observed more frequently among young women (Table 2).

Coronary angiography findings and revascularization strategies

Seven out of eight young women admitted for evolving STEMI (<12 hours) underwent thrombolysis, resulting in three unsuccessful cases that later underwent rescue coronary angiography.

Percutaneous coronary intervention (PCI) was performed on 28 young women (64%) compared to 125 (81.7%) among the older women. In contrast, medical treatment was recommended for 16 young patients.

Single-vessel disease (SVD) was found in 25 young women (57%), followed by double-vessel disease (DVD) (6, 13%) and triple-vessel disease (TVD) (5, 11%). The left anterior descending artery (LAD) was the most commonly affected coronary artery. Young women showed a higher incidence of normal coronary angiography compared to older women. In addition, SCAD was significantly more prevalent among young women compared to older women (Table 3).

**Table 3:** Comparison of angiography findings and management. STEMI: ST elevation myocardial infarction; NSTEMI: non-ST elevation myocardial infarction; PCI: percutaneous coronary intervention; RCA: right coronary artery; SCAD: spontaneous coronary artery dissection; CABG: coronary artery bypass grafting.

	Group ≤ 50 years old (N = 44)	Group > 50 years old (N = 153)	p-value
Initial management			
Median time to coronary angiography			
STEMI (h)	23 ± 16.6	39.3 ± 56.9	0.053
NSTEMI (h)	49 ± 26.8	60.9 ± 45.3	0.067
Primary PCI	8 (18%)	35 (22.9%)	0.01
Thrombolysis	7 (16%)	19 (12.4%)	0.659
Success	3/7 (43%)	11/19 (57.9%)	0.864
Rescue angioplasty	4/7 (57%)	8/19 (42.1%)	0.864
Angiographic findings			
Normal coronary angiography	8 (18%)	12 (7.8%)	0.084
Coronary status			
Single-vessel disease	25 (57%)	48 (31.3%)	<0.001
Double-vessel disease	6 (13%)	58 (37.9%)	0.006
Triple-vessel disease	5 (11%)	35 (22.9%)	0.162
Involved coronary arteries			
Left main	0 (0%)	13 (8.5%)	0.077
Left anterior descending	24 (55%)	122 (79.9%)	0.002
Left circumflex	13 (30%)	61 (39.9%)	0.213
Right coronary	15 (34%)	82 (53.6%)	0.023
Revascularization strategies			
Percutaneous Intervention	28 (64%)	125 (81.7%)	0.011
CABG	0 (0%)	8 (8.2%)	0.203
Medical treatment only	16 (36%)	20 (13.1%)	0.001
Spontaneous coronary artery dissection			
Number of patients	7 (16%)	4 (2.6%)	0.001
Culprit artery			
Left anterior descending	5 (11%)	2 (1.3%)	0.007
Left circumflex	2 (5%)	0 (0%)	0.049
Right coronary	0 (0%)	2 (1.3%)	0.602
Type of SCAD			
Type 1	1 (2%)	1 (0.7%)	0.427
Type 2	6 (14%)	3 (2%)	0.427
Type 3	0 (0%)	0 (0%)	-
Type 4	0 (0%)	0 (0%)	-
Treatment of SCAD			
Percutaneous intervention	3 (7%)	2 (1.3%)	0.075
Medical treatment only	4 (9%)	2 (1.3%)	0.023

Etiological assessment of myocardial infarction

Cardiac MRI was incorporated as part of the etiological assessment of MI cases presenting with normal coronary angiography. Atherosclerosis emerged as the predominant etiology, affecting 29 young women, but lower than the prevalence observed in older women (66% vs. 90.2%, p < 0.001). Furthermore, young women had a higher incidence of SCAD. For the remaining MI etiologies, no significant disparities were observed between the two groups (Table 4).

**Table 4:** Comparison of myocardial infarction etiologies. MINOCA: myocardial infarction with non-obstructive coronary artery disease; SCAD: spontaneous coronary artery dissection.

	Group ≤ 50 years old (N = 44)	Group > 50 50 years old (N = 153)	p-value
Atherosclerosis	29 (66%)	138 (90.2%)	<0.001
MINOCA	15 (34%)	15 (9.8%)	<0.001
SCAD	7 (16%)	4 (2.6%)	0.001
Myocarditis	3 (7%)	2 (1.3%)	0.075
Transmural infarction with normal coronaries	3 (7%)	7 (4.6%)	0.696
Takotsubo syndrome	1 (2%)	1 (0.7%)	0.398
Prinzmetal syndrome	1 (2%)	1 (0.7%)	0.398

A more precise analysis revealed that MI with non-obstructive coronary artery disease (MINOCA) was significantly more prevalent among young women compared to older women (p < 0.001).

In-hospital and long-term outcomes

The median length of hospitalization among young women was eight days. Young women were less likely to experience MACCE during their hospital stay compared to older women. However, older women tended to have left heart failure. In-hospital mortality was higher in the group of older women than in young women, although the difference was not statistically significant (p = 0.341) (Table 5).

**Table 5:** Comparison of in-hospital outcomes. MACCE: major cardiac and cerebrovascular events.

	Group ≤ 50 years old (N = 44)	Group > 50 years old (N = 153)	p-value
Follow-up median (/months)	58.2 ± 29.9	45.5 ± 26.4	0.118
MACCE	5 (11%)	50 (32.7%)	0.005
Mortality	1 (2%)	12 (7.8%)	0.305
Angina	3 (7%)	20 (13.0%)	0.240
Percutaneous coronary intervention	2 (5%)	21 (13.7%)	0.031
Myocardial Infarction	2 (5%)	24 (15.7%)	0.122
Stroke	0 (0%)	3 (1.9%)	0.460

On the long-term follow-up, only one death (2%) was recorded among the younger women after hospital discharge. The older women showed higher mortality, but the difference was not statistically significant.

Only five young patients (11%) experienced MACCE during the follow-up period compared to 50 older women (32.7%) (Table 6).

**Table 6:** Comparison of follow-up outcomes. MACCE: major cardiac and cerebrovascular events.

	Group ≤ 50 years old (N = 44)	Group > 50 years old (N = 153)	p-value
Follow-up median (/months)	58.2 ± 29.9	45.5 ± 26.4	0.118
MACCE	5 (11%)	50 (32.7%)	0.005
Mortality	1 (2%)	12 (7.8%)	0.305
Angina	3 (7%)	20 (13.0%)	0.240
Percutaneous coronary intervention	2 (5%)	21 (13.7%)	0.031
Myocardial infarction	2 (5%)	24 (15.7%)	0.122
Stroke	0 (0%)	3 (1.9%)	0.460

Survival data

**Figure 1:**
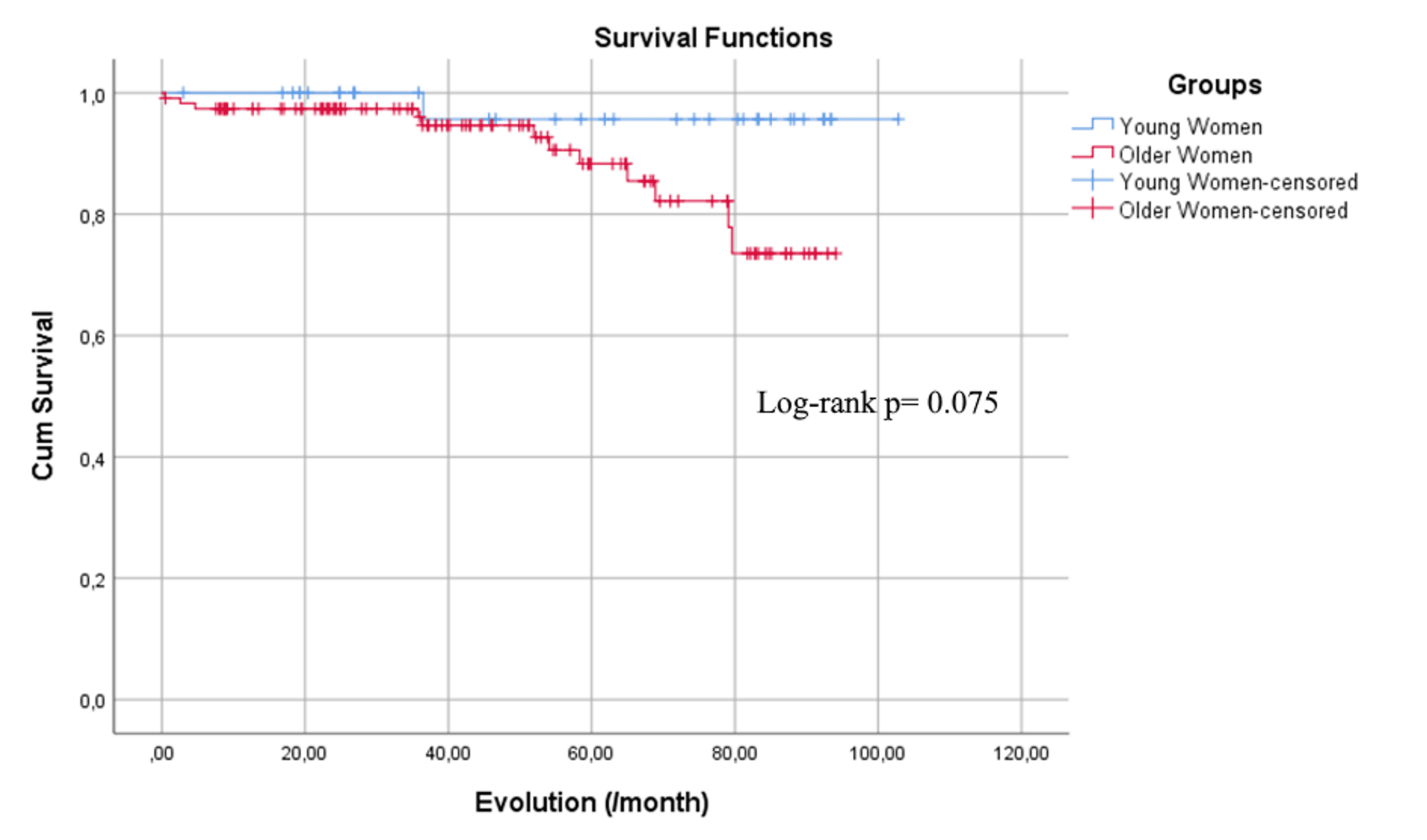
The Kaplan-Meier survival curve.

**Figure 2:**
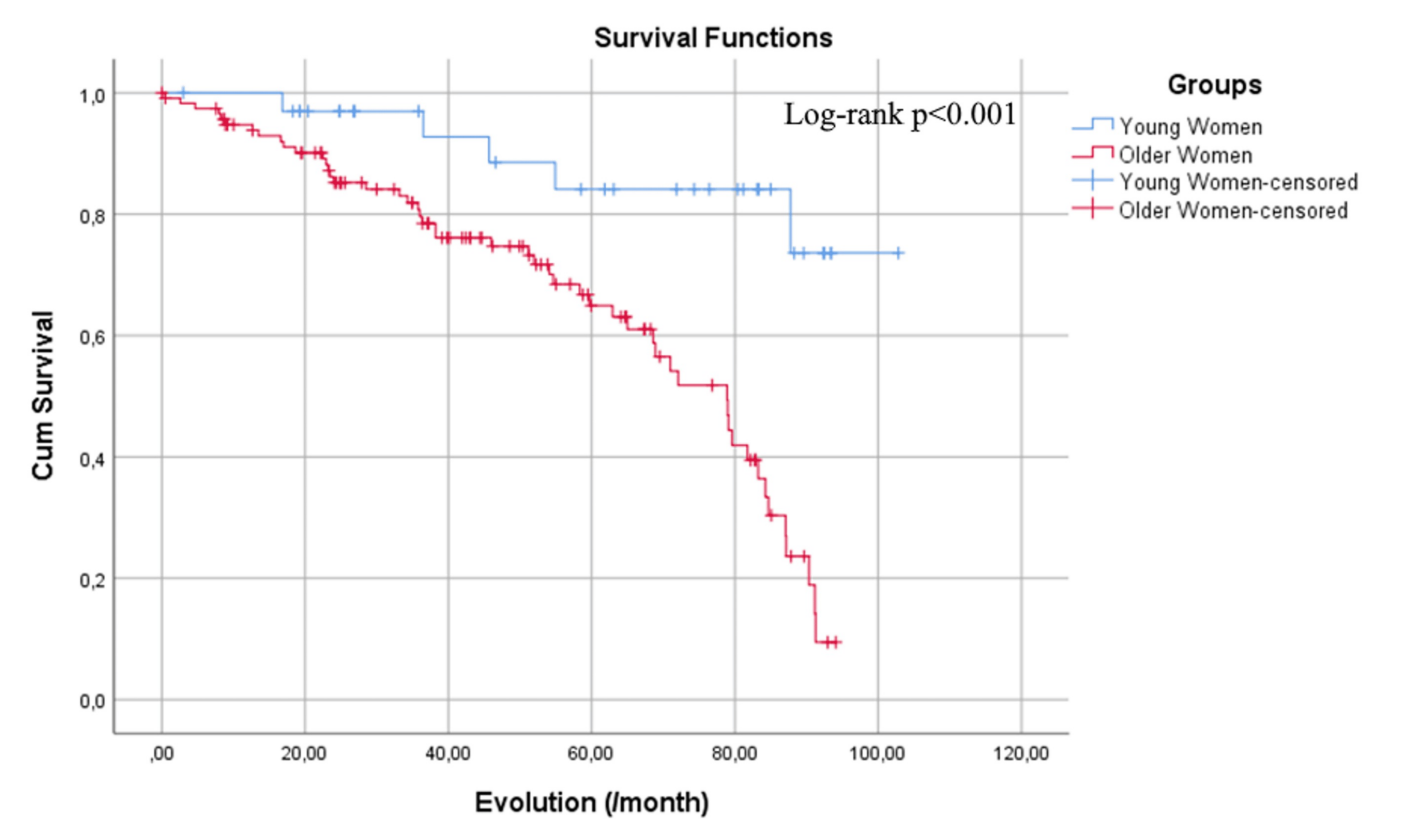
The Kaplan-Meier event-free survival.

The analysis of survival curves showed that young women had a mean survival time of 99.9 months, while older women had a slightly lower mean survival time of 84.7 months, which was not statistically significant (p = 0.075) (Figure 1). Conversely, the Kaplan-Meier survival curve analysis for MACCE demonstrated that young women had an event-free survival time of 91.3 months. In contrast, older women showed a significantly lower event-free survival time of 65.5 months (p < 0.001) (Figure 2).

## Discussion

In the case of young women, the occurrence of acute coronary syndrome (ACS) is a rare event. However, when examining hospitalization rates for MI, the proportion of young patients (aged 35-54 years) appears to be increasing, particularly among young women [1]. In our study, the age limit was set at 50 years. This age limit has been widely adopted by numerous authors in the literature [6,7]. This variation in findings may be attributed to the criteria used to define "young women." The age limit used to define young coronary patients should theoretically differ between men and women since the peak incidence of coronary disease in women occurs approximately 10 years later than in men [8].

Previous studies have consistently shown that young women with MI have multiple CVRFs, as found in our study [1,9]. Hypertension, the most common CVRF in the study, affected 53% of young women. However, it showed a significantly higher prevalence in older women, which was consistent with the findings of Bęćkowski et al. (p < 0.0001) and the YOUNG-MI registry [7,10]. On the other hand, smoking was the most remarkable CVRF among young women (46%) compared to older women. High rates of smoking in young women were found in previous studies ranging from 55.5% to 80.3% [7]. Bęćkowski et al. showed that the rate of smokers was higher among young women (48.7% vs. 22.2%, p < 0.0001) [10]. All studies agree that smoking is the main CVRF in young women, promoting atherosclerosis and accelerating the progression of atherosclerotic plaques, and it increases the risk of STEMI in young women by more than eight times compared to non-smokers [11]. Diabetes was found in 44% of young women. Despite the variety of results reported in the literature ranging from 10.6% to 23.8% [6,7,10], diabetes is still considered the CVRF most associated with an increased risk of MI in women up to eight times higher than in non-diabetic women [12]. A total of 5% of young women were obese. The high prevalence of obesity and overweight in young women with coronary artery disease (CAD) has been highlighted in several studies, including 43.9% in the YOUNG-MI registry and up to 53.7% in the study by Lu et al. [7,13]. A family history of CAD was more prevalent in young women. The prevalence of a family history of CAD in young women is high, ranging between 28.5% and 35% [6,7,13]. In fact, a family history of CAD is a major risk factor for MI in young women, with a four-fold increase in risk [14].

In addition to CVRF, young women also have a higher prevalence of comorbidities, such as chronic lung disease and autoimmune diseases. The involvement of autoimmune diseases in the development of early coronary events in young women has been reported in the literature [15]. The pathophysiological mechanism is still not fully understood, but it is clear that controlling the underlying disease is essential [16].

A high rate of early menopause was found among young women. The WAMIF study found that 15% of women aged below 40 years old with MI were menopausal [6]. Lu et al. also reported that 9.2% of young women with MI were menopausal before the age of 45 years [13]. Early menopause has been associated with the occurrence of MI in young women and the risk of MI is higher if menopause occurs before the age of 45 years [17].

The clinical presentation of MI in women has traditionally been described as atypical [18]. However, this notion is being challenged by recent studies, particularly in young women. In our study, the most common presenting symptom was chest pain in both groups. Young women also tended to present with more associated symptoms, such as dyspnea, nausea, and vomiting. Similar results have been reported in the Polish study by Bęćkowski et al., showing that 90.4% of young women with MI presented with chest pain, compared to 88.5% of older women (p = 0.025) [10]. The WAMIF study, YOUNG-MI study, and VIRGO study also found that chest pain was the most common presenting symptom in young women, with rates of 90.6%, 90%, and 87%, respectively [6,7,19].

Young women were more likely to present with NSTEMI than older women. The VIRGO study showed the same rate among young women (52.1%) [19]. However, Bęćkowski et al. found that STEMI was the dominant type of ACS in the younger cohort (42% vs. 26.1%) [10]. This contrast in data may be elucidated by the tendency of young women in our cohort to present with minor infarctions, confirmed by the elevated prevalence of MINOCA, which is recognized for presenting as NSTEMI, as evidenced in the literature [20].

The majority of young women presented late (> six hours from the onset of symptoms). Indeed, young women are more likely to present late and frequently after six hours of symptom onset [21]. The reasons for this delay are likely multifactorial, including the fact that young women may not recognize the symptoms of ACS as being serious [22]. The VIRGO study [23] showed that more than 40% of young women did not consider chest pain as a sign of ACS. This highlights a significant gap in the recognition of heart disease in young women who are generally considered a low-risk population.

Significant differences were observed in the revascularization strategies for STEMI between the two groups, as thrombolysis was the most common revascularization strategy in young women. This may be explained by the fact that almost half of the young women admitted for STEMI consulted within eligible time frames for thrombolysis (<12 hours) and that young women are less likely to have contraindications to thrombolysis [24]. The lack of resources for primary PCI in our center may also have contributed to the higher use of thrombolysis in young women.

In terms of angiographic findings, Bęćkowski et al. and Ezhumalai et al. demonstrated that SVD was more prevalent in young women with obstructive CAD (55% vs. 40.9% and 38.3% vs. 24.4%) [10,25]. The studies also revealed higher rates of normal or non-significant lesions in coronary angiography among young women compared to their older counterparts (29.9% vs. 17.3% and 26.4% vs. 19.3%, respectively), aligning with our own findings. In addition, the analysis of the French WAMIF registry showed that TVD was present in only 29.6% of young women [6]. In fact, young women are more likely to have a less severe form of CAD than older women and tend to present with MINOCA, leading to a higher prevalence of normal coronary angiography [26].

Atherosclerosis was the most common cause of MI in both groups with higher rates in older women, a conclusion also seen in Ezhumalai et al.'s study with 35.4% of women under age 55 having atherosclerotic MI compared to 55.9% of older women (p < 0.0001) [25]. However, younger women are more likely to have MINOCA, especially SCAD. SCAD is a rare condition that accounts for less than 1% of cases [27]. It is most common in women aged 47 to 53 years [27]. Cohort studies with direct angiographic analysis have shown that one-quarter to one-third of women presenting with MI under the age of 50 are caused by SCAD [28].

The prevalence of transmural MI with normal coronaries was higher in young women than in older women. It is recognized as a transmural MI of atherosclerotic origin without significant coronary stenosis due to the rupture of an atherosclerotic plaque [29]. The classic demographic profile of these patients includes young women who smoke but have few CVRFs [29].

The in-hospital outcome was favorable for young women, attributed to a lower prevalence of comorbidities, fewer CVRF, and less extensive atherosclerotic disease. As expected, we observed a higher incidence of MACCE in older women, and this difference was statistically significant, primarily driven by the occurrence of acute left heart failure, in alignment with existing literature [10]. Contrastingly, our study revealed no in-hospital deaths among young women, compared to a mortality rate of 3.9% in older women. Lower mortality is reported among younger women with MI than in older women [30]. Age emerges as a significant risk factor for both in-hospital mortality and MACCE.

On long-term follow-up, the prognosis was good among young women. Several studies have drawn the same conclusion that young women have a favorable prognosis compared to older patients [10,30].

Strength and limitation

To our knowledge, this is the only published Tunisian series evaluating young female patients with MI. The study examines multiple variables, including epidemiological, clinical, biological, echocardiographic, angiographic, and evolutionary aspects. The inclusions were exhaustive, reflecting a real-life registry and providing a realistic view of the typical management of our patients. Additionally, the long-term follow-up of our patients allows for the evaluation of their prognosis in terms of mortality through survival analysis using the Kaplan-Meier method.

The main limitations of our study include its monocentric nature, the small sample size resulting in a limited number of cardiac events, and consequently, reduced study power. Additionally, the retrospective design and the limited duration of inclusions may impact the scope of the results. Moreover, the absence of intracoronary imaging techniques in our center, which are sometimes necessary for a comprehensive etiological assessment of ACS with normal coronary angiographies, represents another limitation.

## Conclusions

MI remains a critical diagnostic and therapeutic emergency, increasingly prevalent in developing countries and affecting a younger demographic due to contemporary lifestyle changes and heightened exposure to risk factors. Despite being traditionally considered low-risk, young women with MI present unique challenges and characteristics compared to older counterparts. They show a high burden of CVRFs and comorbidities, including early menopause, which significantly contributes to their heightened risk of coronary events. Clinical presentations often differ, with delayed hospital presentations posing diagnostic and therapeutic challenges. Our study focused on the rare entity of MI in young women, unraveling its distinct characteristics through a comprehensive retrospective analysis of epidemiological, clinical, echocardiographic, therapeutic, and prognostic variables. Further research is essential to better understand the underlying mechanisms and optimize prevention and treatment strategies for this vulnerable population.
